# Impact of out-of-pocket health expenditure on poverty in India

**DOI:** 10.3389/fpubh.2026.1762886

**Published:** 2026-03-19

**Authors:** Neeraj Soni, Rajabushan Jagadish Nayak, Prasant Blon, Meghna Ghosh, Kanupriya Singh, Ashish Singh

**Affiliations:** 1 Sri Sathya Sai Institute of Higher Learning (Deemed to be University), Prasanthi Nilayam, India; 2 BITS Law School, Mumbai, India; 3 Independent Health Researcher, Mumbai, India; 4 Shailesh J Mehta School of Management, Indian Institute of Technology Bombay, Mumbai, India

**Keywords:** health, India, out-of-pocket health expenditure, poverty, universal insurance coverage

## Abstract

As per the World Bank Poverty and Inequality Platform (2021), approximately 12.92% of India’s population was below the poverty line in 2018, based on the international poverty line of $2.15 per day (PPP 2017). According to the National Health Authority (NHA) Estimates for 2018–19, the out-of-pocket health expenditure (OOPHE) share of total health expenditure (THE) was reported as 48.2% for the entire population, a decline from 64.2% in 2013–14. Thus, OOPHE is a major reason pushing households into poverty. Therefore, we seek to estimate the impact of OOPHE on poverty in India. We use data from the nationally representative survey on Household Social Consumption Health (2017–2018) and estimate poverty gap at the household level before and after making the OOPHE. Further, binary logit model was used for predicting the effects of various factors on the incidence of poverty. We find that the duration of hospitalization, household size, usage of private healthcare, out of pocket health expenditure, income, aged people in the family and caste are the significant determinants of impoverishment. Also, the implementation of various healthcare schemes and public health initiatives have not been substantial as they are found to be limited, and a significant proportion of the population is left with no health coverage. Findings stress on the need for the expansion of public health insurance coverage for safeguarding households against falling into poverty.

## 1 Introduction

One of the primary objectives of the health system in the modern age, in general, has been assuring that households are safeguarded from the financial risks associated with health expenditure. Thus, India has been taking an active role in ensuring that all the citizens have access to Universal Health Coverage. According to the Sustainable Development Goals (2000) too, health has been given special significance, and it has been emphasized as a dimension of poverty. According to the National Health Profile (1), in India, out-of-pocket expenditure on healthcare constitutes around 63% of total healthcare spending, reflecting the dominance of OOPHE in healthcare financing. It also highlights that the average out-of-pocket per capita expenditure on health in India is approximately ₹2,394 ($32), which is significantly higher compared to the global average. As per the NSSO 75th round (2017–18) report (2), high out-of-pocket spending is particularly burdensome for rural households as 68% of rural and 58% of urban healthcare expenditure is estimated as out-of-pocket. The report also indicates that around 16.8% of households in India faced the financial strain of catastrophic health expenditures due to OOP expenditure.

In the Indian healthcare landscape, out-of-pocket health expenditure stands as a formidable barrier to equitable access to essential services, often entrenching families into poverty, or increasing the intensity of poverty. By using the methodology of the Suresh Tendulkar Committee report (2009), out of 1210.19 million population in India (Census 2011), almost 269 million, i.e., 21.9% of the population were below the poverty line. Academic research has found that high out-of-pocket health expenditure pushes the households into poverty. Around 39 million Indians were pushed into poverty in 1999–2000 due to out-of-pocket health expenditures (3). Various literature shows us that out-of-pocket health expenditure can increase the incidence of poverty and can widen the normalized poverty gap; additionally, it has a negative impact on equity (4, 5).

As per the World Bank Poverty and Inequality Platform, approximately 11.09% of India’s population is below the poverty line. Also, of the total health expenditure, 48.2% consists of out-of-pocket health expenditure (OOPHE) or the population in poverty, which implies that India has one of the highest OOPHE in the world. Thus, OOPHE is a major reason for pushing households into poverty. This discussion can also be seen in the light of the debate on distress financing (6–8) where up to 80% of the household income/savings gets used against health expenditure along with substantial borrowings as well as sale of household assets.

The prevalence of high out-of-pocket health expenditure in India poses significant challenges to the affordability, accessibility, and equity of healthcare services. The increasing out-of-pocket expenditure has not only affected the households by pushing them into poverty but has also increased the incidence of poverty. Out-of-pocket health expenditure can hinder efforts to reduce poverty, as rising healthcare costs may push not only the already vulnerable but also households that are not near the poverty line into poverty. Thus, it becomes very important to delve on the intricacies of OOPHE in India by looking into its causes, consequences, and potential solutions. Thus, this research focuses on finding the impact of OOPHE on poverty and additionally finding the factors affecting the OOPHE. The objectives of our research are:

(i) To analyze the relationship between the incidence of poverty and OOPHE
(ii) To find the determinants of incidence of poverty after making OOPHE.

The most recent national survey by the National Sample Survey Organization (2017–2018) has been used to extract household-level data for analyzing the impact of OOPHE on household poverty. Given this context, this study aims to analyze the impact of out-of-pocket health expenditures on poverty in India, with a focus on how healthcare-related financial burdens influence poverty incidence and depth, particularly among vulnerable populations. While doing so, we not only considered the national level poverty lines but also the state level poverty line which is a departure from the existing scholarship and a novelty of our study. Further we have adjusted the poverty lines for inflation. In addition, our estimates are recent as we have used the latest survey for the estimation.

## 2 Materials and methods

### 2.1 Data source

The data for this study has been extracted from the National Sample Survey Organization. The National Sample Survey Office (NSSO) is a key statistical agency in India responsible for conducting large-scale surveys and collecting socio-economic data. We have used the Household Social Consumption: Health data, NSS 75th Round Schedule (2017–2018) for our analysis. The survey collected a random sample of 1,13,823 households, out of which 64,552 were rural households and 49,271 households were urban households. The total number of individuals surveyed was 5,55,115, covering all the states and union territories. The poverty line threshold which is used in this study for calculating poverty is taken from Suresh Tendulkar Committee report (9). Poverty gap is estimated at a household level post OOPHE. The choice of using Tendulkar Committee recommendations for poverty lines for estimation is guided by—first, the Government of India itself is following Tendulkar Committee recommendations for poverty estimation [Sabha (10) can be seen in this regard]; and second, the existing scholarship on the subject is also using Tendulkar Committee recommendations to a large extent [Sriram & Albadrani (11) and the references therein].

### 2.2 Methodology

To evaluate the impoverishing effects of OOPHE, we use two key poverty metrics: the poverty headcount ratio and the poverty gap index. These indices help us to understand not just how many people are pushed into poverty due to healthcare payments, but also how deeply they are affected. The healthcare payments include payment against—medical advice, package fee, Doctor’s/Surgeon’s fee, medicines, diagnostic tests, bed charges, transportation for patients etc.^1^ Since there is no officially updated poverty line available for the year 2017–18, we rely on the poverty thresholds recommended by the Tendulkar Committee, in line with findings from earlier studies (9, 10). These state-specific poverty lines for rural and urban regions have been adjusted for inflation to reflect the 2017–18 price levels, aligning with the reference period of the survey data. Thus, for the purpose of calculating poverty measures in this study, we use inflation-adjusted poverty lines derived from the Tendulkar Committee’s recommendations to ensure consistency and comparability across states and over time. To calculate the impact of OOPHE on health on poverty, we will calculate the poverty rates before and after subtracting OOPHE from each household’s consumption expenditure. Equation 1 calculates Pre-OOPHE poverty headcount rate (*Pre Hp*):


$$\mathrm{Pre}\;\mathrm{Hp}=\frac{1}{n}\Sigma \;(x_{i}\leq \mathrm{PL})$$ (1)

Where $x_{i}$ is the per capita consumption expenditure of an individual (in Rupees), PL is the poverty line (in Rupees), n is the total number of individuals. This Equation 2 calculates Post-Payment poverty headcount ratio (Post Hp) which captures the additional burden of healthcare payemnts (Post Hp):


$$\text{Post}\;\mathrm{Hp}=\frac{1}{n}\Sigma \;((x_{i}-\text{OOPE})\leq \mathrm{PL})$$ (2)

Where OOPHE is the out of the pocket expenditure on health by the individual i. Equation 3 measures the number of people newly pushed into poverty after OOPHE.


NewHp=PostHp–PreHp (3)

If the OOPHE is positive in Equation 2, Post Hp would be greater than the Pre Hp. New Hp is the additional number of people who enter poverty after incurring OOPHE.

To assess the depth of poverty, We calculate the poverty gap in Equation 4, which reflects how far below the poverty line poor individuals fall, on an average. The gap index is defined as:


$$\text{Poverty}\;\mathrm{Gap}=\frac{1}{n}\Sigma \;(\mathrm{PL}-x_{i})\;\text{for}\;x_{i}\leq \mathrm{PL}$$ (4)

This gives the average shortfall in consumption expenditure among those who are already poor.

A logit regression model has been used to study the impact of various factors that may possibly lead an individual to poverty due to out-of-pocket healthcare expenditure. The difference between the average level of poverty before and after the OOPHE is measured as effect of OOPHE on poverty. As a result, the household count ratio has been used to calculate the poverty gap. Additionally, the marginal effects in the logit models have been used to capture the change in the probability of the dependent variables due to change in the independent variable holding other independent variables constant. The marginal analysis provides significant information as to how the change in the independent variables affects the likelihood of the binary outcomes.

Thus, a dichotomous variable is generated to represent the occurrence of impoverishment within the household. A value of 0 is assigned to the household that does not fall below the poverty line after making out-of-pocket expenditure on healthcare, on the other hand, a value of 1 is assigned to the household that does experience impoverishment. Consequently, this binary variable serves as both the dependent variable in the logit regression model and a measure of the prevalence of poverty in the household. Table 3 explains the variables used for the study and its descriptive statistics.

## 3 Results

Using the state-wise poverty thresholds based on the Tendulkar methodology for Monthly *Per Capita* Expenditure (MPCE), and appropriately adjusted for inflation, the estimated pre-OOPHE poverty rates stand at 17.71% in rural areas, 6.80% in urban areas, and 12.26% at the aggregate level. Out-of-pocket health expenditures impose an additional, and often uncertain, financial burden on households, potentially exacerbating their vulnerability to poverty (6, 12). Accordingly, a key objective of this study is to quantify the impact of OOPHE on poverty incidence. As highlighted in Table 1, when OOPHE is subtracted from household income, the resulting post-OOPHE poverty rates rise to 19.05% in rural areas, 7.97% in urban areas, and 13.51% overall—indicating an increase of 1.34, 1.17, and 1.25%, respectively.

**Table 1:** Incidence of poverty.

Sector	Pre-OOPHE poverty rate (%)	Post-OOPHE poverty rate (%)	Total difference (%)
Rural	17.71	19.05	1.34
Urban	6.80	7.97	1.17
Overall	12.26	13.51	1.25

This increase of 1.25% in the poverty headcount corresponds to 3.1 million households falling into poverty. Thus, it highlights the pressing need for affordable and accessible healthcare facilities to alleviate the financial burden on households across the nation. Finding a more equitable healthcare solution and addressing the underlying causes of high out-of-pocket costs are crucial if we are to guarantee that everyone has access to healthcare without running the risk of falling into poverty. Further, we can observe that between rural and urban areas, increase in the poverty rates in the rural areas is more compared to the urban areas. In rural areas, the poverty rate, pre-OOPHE was 17.71% and post-OOPHE was 19.05%, but in the urban areas, we can see that the poverty rate before OOP health expenditure was 6.80%, which after OOPHE payments, increased to 7.97%. The poverty gap in the rural sector stands at 1.34% whereas the urban sector poverty gap stands at 1.17%. We can observe that the occurrence of poverty has increased after making the out-of-pocket healthcare expenditures, both in the rural and urban areas. It is also observed that in the rural areas, the incidence of poverty has been more than in the urban areas, as there has been an increase in the migration from rural to urban areas (13). The living condition of those migrants has not improved because of low employment opportunities in the urban areas, almost 27% of the urban population lives under the poverty line (14), thus leading migrants to deplorable living conditions. Thus, having a low income makes workers vulnerable to high health expenditure, leading to OOPHE.

**Figure 1:**
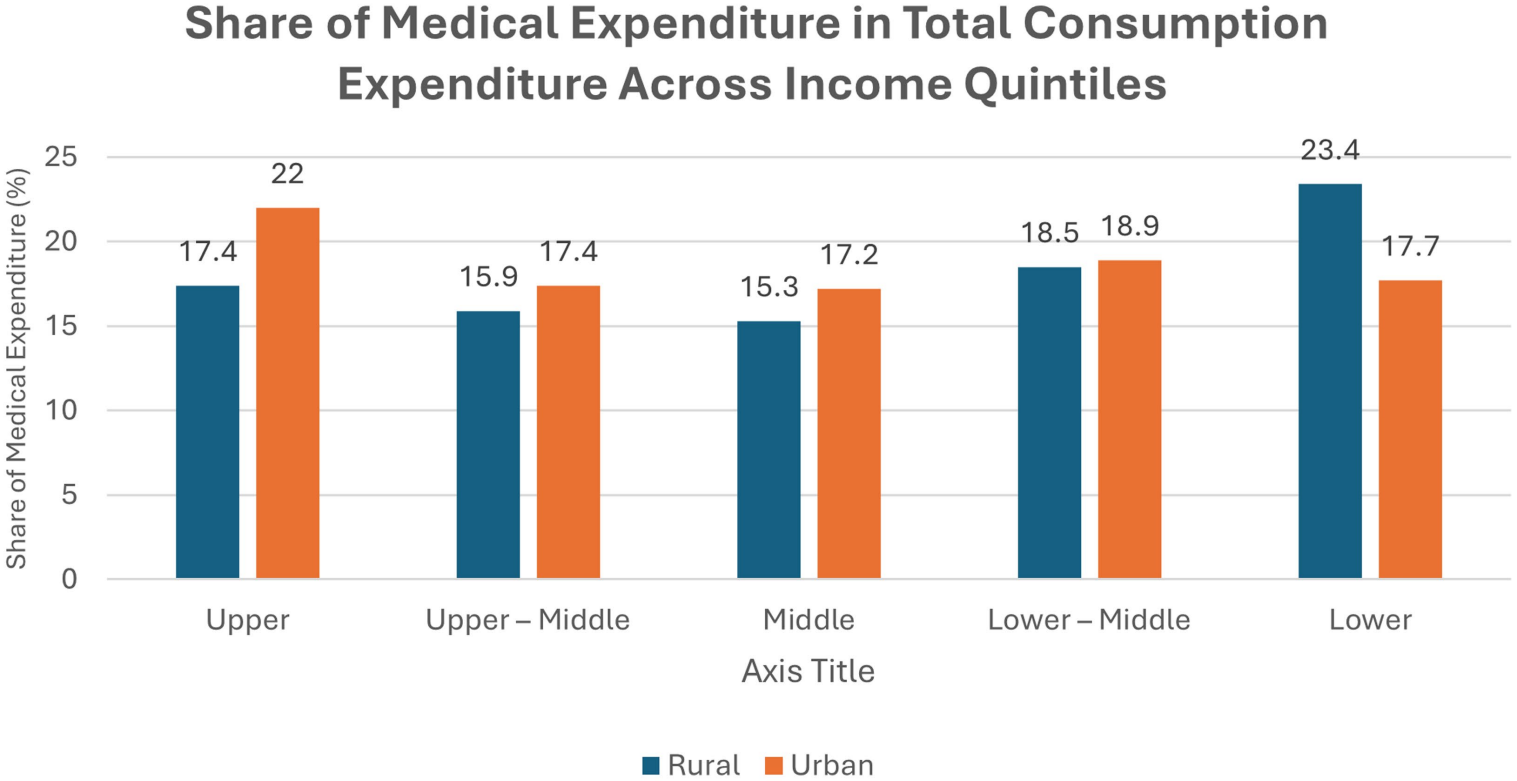
Share of medical expenditure in total consumption expenditure across income quintiles.

Figure 1 presents a comprehensive overview of the proportion of total medical expenditure to total consumption expenditure across different income quantiles in both rural and urban areas. The data in Table 2 reveals striking disparities in the burden of out-of-pocket expenditure (OOPHE) on health, highlighting how medical expenses can significantly strain household incomes, particularly among the lower-income groups and in some states.

**Table 2:** Share of medical expenditure in total consumption expenditure across income.

State	Upper		Upper–Middle		Middle		Lower–Middle		Lower	
	Rural (%)	Urban (%)	Rural (%)	Urban (%)	Rural (%)	Urban (%)	Rural (%)	Urban (%)	Rural (%)	Urban (%)
Jammu and Kashmir	9.00	17.80	6.20	8.80	3.20	5.50	4.20	9.90	4.20	8.00
Himachal Pradesh	31.10	27.60	14.10	16.70	18.50	10.40	13.10	12.70	36.40	11.70
Punjab	19.10	17.10	35.70	8.80	11.30	15.60	20.00	13.00	20.10	14.30
Uttarakhand	14.50	24.80	9.60	8.80	9.50	8.80	11.00	11.80	14.10	14.20
Haryana	15.30	45.80	20.50	12.80	10.10	20.20	16.00	23.40	11.70	11.90
Rajasthan	15.70	7.60	13.10	10.30	11.10	10.00	13.10	11.60	32.60	14.40
Uttar Pradesh	19.80	21.40	19.60	18.60	16.50	18.30	19.30	33.00	31.20	18.20
Bihar	8.50	9.60	7.80	11.00	11.60	11.80	11.90	7.90	86.10	15.60
Sikkim	4.70	10.90	6.70	1.90	5.70	9.00	5.00	7.40	9.60	5.30
Arunachal Pradesh	9.60	9.90	7.50	6.20	4.30	4.60	3.30	5.90	4.20	4.10
Nagaland	13.00	8.90	5.20	13.40	5.80	5.00	3.40	6.20	5.00	6.30
Manipur	9.30	12.40	9.00	10.00	8.80	13.50	16.10	13.60	10.40	13.90
Mizoram	6.60	16.10	5.20	5.20	6.90	8.20	4.90	6.20	3.30	5.50
Tripura	6.60	167.00	6.10	5.80	5.40	9.20	6.40	8.90	6.10	13.70
Meghalaya	2.10	1.10	2.10	2.80	3.10	14.10	2.60	8.40	1.00	7.60
Assam	8.30	29.30	5.80	14.60	7.10	11.00	11.10	22.60	22.00	18.90
West Bengal	29.60	14.30	12.00	12.20	14.00	13.00	17.50	16.40	41.10	22.20
Jharkhand	9.40	30.60	16.30	26.30	15.60	13.30	15.40	25.80	22.90	14.60
Odisha	15.80	14.50	19.10	10.70	19.00	13.80	17.50	19.80	16.90	16.10
Chhattisgarh	14.30	22.10	30.90	14.30	13.90	20.10	19.14	19.40	21.20	12.40
Madhya Pradesh	16.10	15.40	15.10	11.90	13.20	14.30	9.10	14.20	17.10	10.30
Gujarat	13.90	37.20	11.90	12.20	10.30	9.70	12.60	14.70	19.20	11.70
Dadara & Nagar Haveli	0.00	0.00	0.60	2.70	0.90	1.00	3.20	6.10	39.30	5.60
Maharashtra	30.10	25.20	20.10	14.90	18.10	17.80	17.00	22.20	16.10	21.60
Andhra Pradesh	26.90	22.00	24.80	18.80	21.10	15.20	22.40	20.40	28.70	18.60
Karnataka	15.60	22.70	12.90	17.70	11.50	11.90	16.00	13.50	7.50	14.30
Goa	65.70	35.10	1.70	5.50	6.50	2.30	4.70	17.80	8.60	18.20
Kerala	20.00	39.50	21.70	38.00	22.80	23.70	20.30	19.70	22.20	21.20
Tamil Nadu	28.60	65.90	19.40	67.10	24.90	35.00	25.60	20.90	21.70	24.20
Puducherry	0.70	33.40	4.80	26.80	30.20	69.70	9.10	14.50	0.80	19.50
Telangana	18.40	68.30	18.40	31.30	21.70	27.30	25.50	21.60	22.80	13.90
Total	17.40	22.00	15.90	17.40	15.30	17.20	18.50	18.90	23.40	17.70

In several states, the proportion of household consumption devoted to medical expenses reaches alarming levels, especially among the poorest segments. For instance, in Bihar’s rural lowest income quantile, households spend an extraordinary 86.1% of their total consumption on medical needs, indicating a catastrophic financial burden. Similarly, in Tripura’s urban lower-middle income group, the share of medical expenditure skyrockets to 167%, suggesting that medical costs far exceed their total consumption expenditure, likely leading to indebtedness or the sacrifice of other essential needs. These extreme cases are not isolated; other states such as West Bengal, Himachal Pradesh, and Tamil Nadu also exhibit high OOPHE percentages among their lower-income populations.

The data further illustrates significant rural–urban disparities. In some states, urban upper-income groups face a higher burden than their rural counterparts, as seen in Tamil Nadu and Telangana, where the urban upper-income quantiles spend 65.9 and 68.3%, respectively. Conversely, in states like West Bengal and Himachal Pradesh, rural lower-income households bear a disproportionately higher burden compared to urban households. These differences may reflect variations in access to public healthcare, insurance coverage, and the availability of affordable medical services across regions (15, 16).

A consistent trend emerges across most states: lower-income groups, both rural and urban, spend a higher proportion of their consumption expenditure on medical needs compared to higher-income groups (17). The national averages reinforce this pattern, with the poorest rural households spending 23.4% of their consumption on health, compared to 17.4% among the richest. However, there are notable exceptions. For example, in Kerala, the urban upper-middle income group allocates a much higher share to medical expenses than other quantiles, highlighting localized inequities in healthcare financing.

Overall, the data underscores the severe strain that OOPHE on health, places on household finances in India. The high percentages, particularly among lower-income and rural populations, suggest that many households are at risk of being pushed into poverty or deepening existing poverty due to medical expenses. This situation is exacerbated in states with weaker public health infrastructure or limited financial protection mechanisms. The findings highlight the urgent need for strengthened public health systems and expanded financial risk protection to reduce the burden of medical expenses on vulnerable populations.

The descriptive statistics provided in Table 3 highlights a detailed profile of 113,823 Indian households, analyzing socio-demographic, economic, and healthcare-related variables. Approximately 48% of households are small (≤4 members), while 45% are medium-sized (5–8 members), and 6% are large (≥9 members). A notable 27% of households include at least one older adult (aged ≥60 years), and 15% report chronic illnesses among members, indicating a substantial caregiving burden. Health insurance coverage remains limited, with only 21% of households having at least one insured member, underscoring reliance on out-of-pocket payments.

**Table 3:** Descriptive statistics.

Variable	Definition	Frequency, *N* = 1,13,823	Percentage %
Household size	Small household (≤4)	55,021	48.34
	Medium household (5 to 8)	51,558	45.30
	Large household (≥9)	7,244	6.36
Age groups (Older Adult)	Inclusion of at least one older adult person aged 60 years or older in the household.	31,194	27.41
Chronic illness	Inclusion of at least one individual with chronic ailment in last 365 days	17,453	15.33
Health insurance coverage	At least one member of the household is covered by the insurance	24,396	21.43
Duration of stay in hospital	≤5 days of stay	55,612	48.86
	6–10 days of stay	18,180	15.97
	11–20 days of stay	5,652	4.97
	≥20 days of stay	2,325	2.04
Usage of a private healthcare facility	Privately maintained medical facilities providing healthcare services in exchange for money.	37,760	33.17
General education	Not literate	26,531	23.31
	Primary education	38,925	34.20
	Secondary education	19,442	17.08
	Higher secondary	14,380	12.63
	Graduate & above	14,545	12.78
Income quantile	Income quantiles		
	Poorest income quintile (reference)	22,765	20.00
	Second income quintile	24,195	21.26
	Third income quintile	21,335	18.74
	Fourth income quintile	22,878	20.10
	Highest income quintile	22,650	19.90
Caste	Others	34,395	30.22
	ST	19,222	16.89
	SC	15,245	13.39
	OBC	44,961	39.50
	Definition and categories	Mean	Std. error
Poverty gap	The ratio by which the mean income of the poor falls below the poverty line		
	Rural	0.164	0.002
	Urban	0.192	0.007
	Overall	0.167	0.002
Reimbursement ratio	Total amount reimbursed/ total medical expenditure		
	Rural	0.019	0.001
	Urban	0.068	0.003
	Overall	0.035	0.001
Out of pocket expenditure	Average out of pocket expenditure by household on medical (in Rs.)		
	Urban	27651.70	664.08
	Rural	14660.13	382.27
	Overall	18761.75	335.90

Hospitalization patterns show that 49% of households experienced short stays (≤5 days), while longer stays (≥20 days) were rare (2%). Private healthcare facilities were utilized by 33% of households, reflecting dependency on paid services despite financial constraints. Education levels vary: 23% of households lack literate members, 34% have primary education, and 13% include graduates, suggesting disparities in health literacy and resource access. Income quintiles are evenly distributed, with 20% in the poorest and 19.9% in the richest categories. Caste composition includes 39.5% OBC, 30.2% General, 16.9% ST, and 13.4% SC households.

Financial metrics reveal a higher urban poverty gap (0.192 urban versus 0.164 rural), indicating greater income shortfalls below the poverty line in cities. Reimbursement ratios for medical expenses are critically low, particularly in rural areas (1.9% versus 6.85% urban), highlighting inadequate insurance support. Average out-of-pocket expenditure (OOPHE) is nearly double in urban households (₹27,652) compared to rural (₹14,660), exacerbating financial strain in cities despite higher average incomes. These patterns collectively illustrate systemic vulnerabilities, where limited insurance coverage, high private healthcare usage, and low reimbursement rates disproportionately affect low-income and urban populations, compounding poverty risks (18).

Results from the logit model (Table 4) shows the impact of various socio-economic variables on the probability of a household entering into poverty after incurring out of pocket expenditure on health. Compared to small households, medium and large households are 27.05 and 59.04 percentage points more likely to fall into poverty, respectively, suggesting that larger households may benefit from shared resources or income pooling. The possible reason could be the division of hospital bills and income pooling of increased number of members. The place where a household is admitted for hospitalization also impacts the OOPHE on health. Households with members admitted to private hospitals increases the probability of entering into poverty by 5.17% pointing to the financial burden of private healthcare (19, 20).

**Table 4:** Logit regression results.

Variables	Category	Marginal effects	St. error	Z
Household size	Small household (reference)			
	Medium household	0.2705	0.0043	0.000
	Large household	0.5904	0.0094	0.000
Caste	Others (reference)			
	SC	0.0415	0.0061	0.000
	ST	0.0729	0.0075	0.000
	OBC	0.0231	0.0049	0.000
Education	Below primary (reference)			
	Primary	−0.0058	0.0049	0.239
	Secondary	−0.0165	0.0065	0.011
	Higher secondary	−0.0162	0.0072	0.024
	Graduate and above	−0.0242	0.0084	0.004
Duration of stay in hospital	Not staying in hospital(reference)			
	Less than 5 days	−0.0361	0.0045	0.000
	5–10 days of stay	0.0265	0.0089	0.000
	11–20 days of stay	0.1455	0.0135	0.000
	20 and above days of stay	0.2655	0.0196	0.000
Income quantile	Poorest income quintile (reference)			
	Second income quintile	−0.3432	0.0061	0.000
	Third income quintile	−0.4662	0.0053	0.000
	Fourth income quintile	−0.4989	0.0051	0.000
	Highest income quintile	−0.5138	0.0049	0.000
Usage of a private healthcare facility	At least one member of the household is admitted to the private hospital	0.0517	0.0056	0.000
Age groups (older adult)	At least one of household is older adult	0.0161	0.0047	0.001
Health Insurance Coverage	At least one is covered under insurance	−0.0378	0.0054	0.000
Out of pocket expenditure	Log of OOPHE on health	0.0202	0.0007	0.000
Constant	Constant	−1.5355	0.0719	0.000

Caste remains a significant determinant of poverty. Compared to the others, SC households are 4.2% more likely, ST households are 7.3% more likely, and OBC are 2.3% more likely to experience poverty. These findings underline the continuing socioeconomic disadvantages faced by historically marginalized communities.

For every unit increase in the log of out-of-pocket health expenditure, the probability of a household becoming poor increases by 2.02 percentage points. This indicates the impoverishing nature of healthcare expenses in India, especially for households lacking adequate financial buffers or insurance coverage (27). Education demonstrates a protective effect—while primary education shows no significant impact, secondary, higher secondary, and graduate education progressively reduces poverty risk, with graduates are 2.4 percentage points less likely to be poor compared to those with education below the primary level. Households with at least one older adult member face a 1.61 percentage point higher probability of entering poverty, reflecting the higher medical needs and financial burden associated with older adult(s) care. This may be because of the additional financial burden associated with older adult(s) care, including medical costs and reduced labor force participation (21).

Households with at least one insured member are 3.78 percentage points less likely to fall into poverty. This highlights the importance of financial protection mechanisms in preventing impoverishment due to health shocks (22, 23). Compared to households with no hospital stay, those with short stays (less than 5 days) are less likely to be poor, whereas stays of 5–10 days, 11–20 days, and over 20 days drastically increase the likelihood of poverty. The data reveal a clear trend: longer hospital stays significantly exacerbate the risk of financial distress. As expected, rising income levels substantially decreases the odds of being poor. Compared to the poorest quintile, the richest households are 51.38 percentage points less likely to fall into poverty after medical spending. This affirms the robustness of income as a determinant of economic well-being. These results are consistent with existing studies (18, 24).

The results of the logit regression suggests that household characteristics such as size, presence of older adult members, chronic illness, and health insurance coverage, significantly influence the likelihood of incurring high out-of-pocket medical expenditure. Despite only 21.4% of households having any health insurance coverage, the low reimbursement ratios-especially in rural areas, indicate that insurance does little to offset costs, leaving most households exposed to financial risk. The use of private healthcare facilities, reported by 33% of households, further increases the probability of higher out-of-pocket payments. The findings highlight that demographic and socioeconomic factors, combined with limited insurance protection and reliance on private care, substantially raise the risk of catastrophic health expenditure for Indian households.

## 4 Conclusion and discussion

This study establishes a relationship between OOPHE and the incidence of poverty within the Indian population. It perhaps provides the latest evidence, and uses not only national poverty lines but also state level poverty lines after adjusting for inflation. In addition, we focus on how healthcare-related financial burdens influence poverty incidence and depth, particularly among vulnerable populations. The increase in poverty among the households after making the OOP expenditure on health is of great concern. The normalized poverty gap of 2.11% has directly impacted approximately 5.24 million households. This trend shows an urgency to mitigate the effects of healthcare expenditure among vulnerable households. Furthermore, our analysis has found many factors that contribute to the increase in the risk of impoverishment. Particularly, the duration of hospitalization, household size, and usage of private healthcare emerge as significant determinants.

The marginal effect analysis displays a glaring reality, as the household size increases, the probability of falling into poverty surges. Similarly, the likelihood of impoverishment has been observed to increase as the duration of hospitalization increases. Thus, these findings reveal a multifaceted nature of challenges posed by OOPHE. Our findings also highlight the impact of out-of-pocket expenditure on different income quintiles. The results reveal the vulnerability of the poor and middle-income groups to impoverishment due to catastrophic expenditure, which is observed by the negative effect of income on probability of falling into poverty. This aligns with established literature on distress financing in India, where lower socioeconomic status strongly correlates with greater reliance on borrowing, asset sales, or external contributions to cope with inpatient expenses, perpetuating cycles of poverty. For example, as per Kastor & Mohanty (6), about 28% of the households incurred catastrophic health expenditure (CHE) and faced distress financing. More than one-third of the inpatients reported distressed financing for heart diseases, neurological disorders, genito urinary problems, musculoskeletal diseases, gastro-intestinal problems and injuries. The likelihood of incurring distress financing was up to 3.2 times higher for those hospitalized. A large proportion of households who had reported distress financing also incurred CHE. Also, Meena & Nayak (8) found that distress financing for inpatient healthcare persists as a major challenge in India with the burden being disproportionately higher among vulnerable groups and in states with weaker public healthcare systems. Moreover, Mishra & Mohanty (25) found that OOPHE had strong economic and educational gradient; with one in four respondents resorting to borrowing or selling to meet the OOPHE. The extent of distress financing was higher in poorer states of Bihar and Odisha; savings was more prevalent among those who met the OOPHE by borrowing/selling of assets. In addition, distress financing was higher among the less educated, poor and in private health centers. The above studies (along with ours) suggest that policymakers should prioritize increasing use of public health centers, targeted insurance coverage, health infrastructure strengthening, need-based financial protection for high-risk groups as well as improving the availability of medicine and diagnostic services which can reduce the extent of distress financing in India.

Post globalization, reforms in India have seen a spike in the growth of private healthcare sector which has a significant risk on impoverishment. Our study has shown that the usage of private healthcare facilities increases the probability of falling into poverty by 13.45%, which highlights the financial burden placed on the household. Our results highlight the importance of health insurance coverage in mitigating the incidence of poverty connected to out-of-pocket healthcare expenditure. Although the negative impact of insurance can be found in our study, the limited coverage across the population poses a challenge. The NSSO’s 75th round on Social Consumption on Health reveals that almost 86% of the population lacks insurance coverage, which suggests an immediate need to expand insurance coverage and enhance financial protection for households. Earlier studies have also reported (similar) impoverishment effect of OOPHE, for example, Ahmad & Mohanty (21) found that 4.3% of the older adult population live below the poverty line before accounting for any healthcare spending but after considering healthcare spending, the poverty headcount rises to 14.2%; Sriram & Albadrani (11) found that, in rural areas, there is an increase of 3.5% in poverty gap after making OOPH payments, whereas, it is 3.6% in the urban areas.

Thus, we can conclude that there is impoverishment due to health care costs despite the existing policies, programs and schemes. Some additional focus on policy intervention to address the complex relationship between healthcare costs and impoverishment might help in this context (26). Also, identifying households who are at a risk of entering into poverty due to out-of-pocket health payments and supporting them through public health initiatives might also be helpful.

Although the approach and the method followed in this study are robust and in line with the scholarship on the subject, the estimates would be sensitive to the choice of poverty lines. Use of alternate poverty lines might lead to different estimates but then it is a reality and challenge of any study on poverty. Since poverty itself depends on the poverty line among other factors, different poverty lines might lead to different estimates.

Lastly, the inadequate budget allocation for the health sector, which stands at around a mere 2% of the total GDP, presents a critical hurdle in addressing the challenges, as highlighted in our study. The World Health Organization recommends that there is a need to address the low budgetary allocation to the health sector and mete out at least 5% of the GDP toward it. The increase in the allocation will be useful in improving the present healthcare system, expanding coverage, and mitigating the risk of impoverishment.

In conclusion, our study highlights the need for comprehensive evidence-based policy reforms to address the challenges posed by OOPHE and its impact on household impoverishment. Thus, a comprehensive, transformative friendly intervention is required to address the intertwined challenges of healthcare access and financial protection.

## Data Availability

Publicly available datasets were analyzed in this study. This data can be found at: Household Social Consumption: Health, NSS 75th Round Schedule-25.0: July 2017–June 2018; https://microdata.gov.in/NADA/index.php/catalog/152#study_desc1684331430702.

## Footnotes

1 The sources of finance include—household income/savings (83.2%), borrowings (9.5%), sale of physical assets (0.3%) and contribution from friends and relatives (7%).
